# MALDI-TOF mass spectrometry as a diagnostic tool in human and veterinary helminthology: a systematic review

**DOI:** 10.1186/s13071-019-3493-9

**Published:** 2019-05-17

**Authors:** Maureen Feucherolles, Sven Poppert, Jürg Utzinger, Sören L. Becker

**Affiliations:** 1 Institute of Medical Microbiology and Hygiene, Saarland University, Homburg/Saar, Germany; 2 Luxembourg Institute of Science and Technology, Environmental Research and Innovation, Belvaux, Luxembourg; 3 Swiss Tropical and Public Health Institute, Basel, Switzerland; 4 University of Basel, Basel, Switzerland

**Keywords:** Diagnosis, Helminths, MALDI-TOF, Matrix-assisted laser desorption/ionization-time of flight, Neglected tropical diseases, Parasites

## Abstract

**Background:**

Matrix-assisted laser desorption/ionization-time of flight (MALDI-TOF) mass spectrometry (MS) has become a widely used technique for the rapid and accurate identification of bacteria, mycobacteria and certain fungal pathogens in the clinical microbiology laboratory. Thus far, only few attempts have been made to apply the technique in clinical parasitology, particularly regarding helminth identification.

**Methods:**

We systematically reviewed the scientific literature on studies pertaining to MALDI-TOF MS as a diagnostic technique for helminths (cestodes, nematodes and trematodes) of medical and veterinary importance. Readily available electronic databases (i.e. PubMed/MEDLINE, ScienceDirect, Cochrane Library, Web of Science and Google Scholar) were searched from inception to 10 October 2018, without restriction on year of publication or language. The titles and abstracts of studies were screened for eligibility by two independent reviewers. Relevant articles were read in full and included in the systematic review.

**Results:**

A total of 84 peer-reviewed articles were considered for the final analysis. Most papers reported on the application of MALDI-TOF for the study of *Caenorhabditis elegans*, and the technique was primarily used for identification of specific proteins rather than entire pathogens. Since 2015, a small number of studies documented the successful use of MALDI-TOF MS for species-specific identification of nematodes of human and veterinary importance, such as *Trichinella* spp. and *Dirofilaria* spp. However, the quality of available data and the number of examined helminth samples was low.

**Conclusions:**

Data on the use of MALDI-TOF MS for the diagnosis of helminths are scarce, but recent evidence suggests a potential role for a reliable identification of nematodes. Future research should explore the diagnostic accuracy of MALDI-TOF MS for identification of (i) adult helminths, larvae and eggs shed in faecal samples; and (ii) helminth-related proteins that are detectable in serum or body fluids of infected individuals.

**Electronic supplementary material:**

The online version of this article (10.1186/s13071-019-3493-9) contains supplementary material, which is available to authorized users.

## Background

In clinical and laboratory diagnostic settings, mass spectrometry (MS) has been utilized for several decades as an approach for protein-centred analysis of samples in medical chemistry [1, 2] and haematology laboratories [3]. In 1975, Anhalt & Fenselau [4] proposed, for the first time, the modification of matrix-assisted laser desorption/ionization time-of-flight (MALDI-TOF) MS as a method to characterize bacteria. Indeed, it was demonstrated that different bacterial species show specific protein mass spectra, which can be used for rapid identification.

During the past decade, MALDI-TOF MS has been widely introduced as a diagnostic technique in microbiology laboratories, where it has replaced most other tools (e.g. phenotypic tests, biochemical identification and agglutination kits) as the first-line pathogen identification method due to its high diagnostic accuracy, robustness, reliability and rapid turn-around time [5]. MALDI-TOF MS is now routinely employed for identification of bacteria [5–8], mycobacteria [5, 9] and some fungi [8]. More recently, MALDI-TOF MS has been applied in research settings for the detection and identification of viruses [10], protozoans and arthropods [11, 12]. In clinical practice, a specific quantity is brought on a target plate (e.g. culture-grown pathogen). Next, the target plate is pre-treated with a chemical reagent (so-called matrix, e.g. α-cyano-4-hydroxycinnamic acid) and subjected to a mass spectrometer for further analysis. The MALDI-TOF apparatus, which is commercially available through different manufacturers [13, 14], uses laser to disperse and ionize the analyte into different molecules, which move through a vacuum driven by an electric field before reaching a detector membrane. The time-of-flight of the various molecules depends on their mass and their electric charge. The specific time-of-flight data are assembled, resulting in specific spectra that are compared to a commercial database, which allows for a rapid identification of the infectious agent and diagnostic accuracy, the latter of which is usually expressed as a score.

MALDI-TOF MS has several strengths if compared to other diagnostic tools, such as polymerase chain reaction (PCR) assays. Once the mass spectrometer and the corresponding databases are available in a laboratory, individual pathogen identification is inexpensive, and the sample preparation procedure does neither require highly skilled technicians nor complex additional laboratory infrastructure. Of note, MALDI-TOF MS is considerably less prone to contamination and results are available within a few minutes. However, constant power supply is a prerequisite, which limits the suitability of the technique in resource-constrained settings. Yet, it should be noted that MALDI-TOF MS is no longer restricted to high-income countries as it is increasingly available in reference laboratories in sub-Saharan Africa and elsewhere [15–19].

MALDI-TOF does not always require culture-grown colonies of a given pathogen. Instead, it can also be employed to identify microorganisms directly from positive blood culture broths [6] with high diagnostic accuracy [7]. Recently, Yang et al. [20] proposed a new framework to analyse MALDI-TOF spectra of bacterial mixtures (instead of only a single pathogen) and to directly characterize each component without purification procedures. Hence, this procedure might become available to be employed directly on other body fluids (e.g. urine, respiratory specimens and faecal samples), which would further increase its relevance in clinical practice [21, 22].

In contrast to clinical bacteriology, little research has been carried out pertaining to the application of MALDI-TOF MS for identification of parasites of human or veterinary importance [23]. Several studies utilized the technique on protozoan parasites such as *Leishmania* spp. [24–26], *Giardia* spp. [27], *Cryptosporidium* spp. [28], *Trypanosoma* spp. [29], *Plasmodium* spp. [30–32] and *Dientamoeba* spp. [33]. These studies used pre-treatment with ethanol and acetonitrile before subjecting the whole pathogens to MALDI-TOF analysis. Additionally, the technique has been used for identification of ectoparasites and vectors, such as ticks [34–37], fleas [38–41] and mosquitoes [42–49]. In contrast to the experiments on protozoans, only selected parts of the ectoparasites and vectors (e.g. legs, thoraxes or wings) were used and subjected to the same extraction method. A further novel approach to apply MALDI-TOF MS in clinical parasitology is the identification of specific serum peptides that are detectable in parasite-infected individuals [50].

Helminth infections caused by nematodes (e.g. *Ascaris lumbricoides*, hookworm, *Strongyloides stercoralis* and *Trichuris trichiura*), cestodes (e.g. *Taenia* spp.) and trematodes (e.g. *Fasciola* spp. and *Schistosoma* spp.) account for a considerable global burden of disease and are among the most common infections in marginalized populations in the tropics and subtropics [51]. Indeed, according to estimates put forth by the Global Burden of Disease (GBD) Study, 3.35 million disability-adjusted life years (DALYs) were attributable to intestinal nematode infections and schistosomiasis in 2017 [52].

Diagnosis is pivotal for effective treatment but requires at least a basic laboratory infrastructure, light microscopes and well-trained laboratory technicians who might not be available in remote areas of tropical and subtropical countries. In high-resource settings, in contrast, knowledge on microscopic identification of helminths is waning in many laboratories. It is surprising that the potential applicability of MALDI-TOF MS as a diagnostic tool for helminths of human and veterinary importance has not yet been systematically assessed, in particular because the technique has been successfully employed for identification of nematode plant pathogens [53–58]. Hence, the goal of this systematic review was to summarize the available data on MALDI-TOF MS application for diagnosis of helminths of medical and veterinary importance, and to provide recommendations for future research needs.

## Methods

### Search strategy

A systematic literature review was performed to identify all relevant scientific studies pertaining to MALDI-TOF MS as a diagnostic identification technique in medical and/or veterinary helminthology. The research was performed according to the guidance expressed in the Preferred Reporting Items for Systematic Reviews and Meta-Analyses (PRISMA) Statement [59].

The following electronic databases were systematically searched: MEDLINE/PubMed, ScienceDirect-Embase, Cochrane Library, Web of Science and Google Scholar. All studies published from inception to 10 October 2018 were eligible for inclusion without language restrictions. The bibliographies of all eligible documents were hand-searched for additional references. Conference abstracts or book chapters detected through these databases and additional library searches were also considered. The search strategy comprised keywords related to the MALDI-TOF MS technique (e.g. “MALDI-TOF” and “matrix-assisted laser desorption/ionization time-of-flight”) and helminthology (e.g. “helminth”, “nematode”, “cestode” and “trematode”). The full search strategies for every database are provided in Additional file 1 and the PRISMA checklist in Additional file 2.

### Eligibility screening

After the systematic literature search, all duplicates were removed. Titles and abstracts of potentially eligible studies were screened to identify manuscripts relevant to the research question. Scientific reports on helminths of either plants or insects as well as studies on symbiotic bacteria of helminths were excluded for this review. However, we kept all publications related to the soil nematode *Caenorhabditis elegans*, as it is used as a model organism for biomedical research. Additionally, studies pertaining to MALDI-TOF/TOF tandem MS were excluded, as this is a different modification of the MALDI-TOF MS technique, which is not routinely employed in clinical microbiology laboratories, but rather in research laboratory use for accurate characterization or sequencing of components like amino acids, metabolites, saccharides, etc. [60–62].

### Data extraction and analysis

The literature search was performed by the first author of this manuscript (MF). All titles and abstracts were then independently reviewed by the first and the last author (MF and SLB) for inclusion and any disagreement was discussed until consensus was reached. All extracted manuscripts were analysed using a reference manager software (Mendeley; http://www.mendeley.com).

## Results

### Search results, number and year of publication of eligible studies

**Fig. 1:**
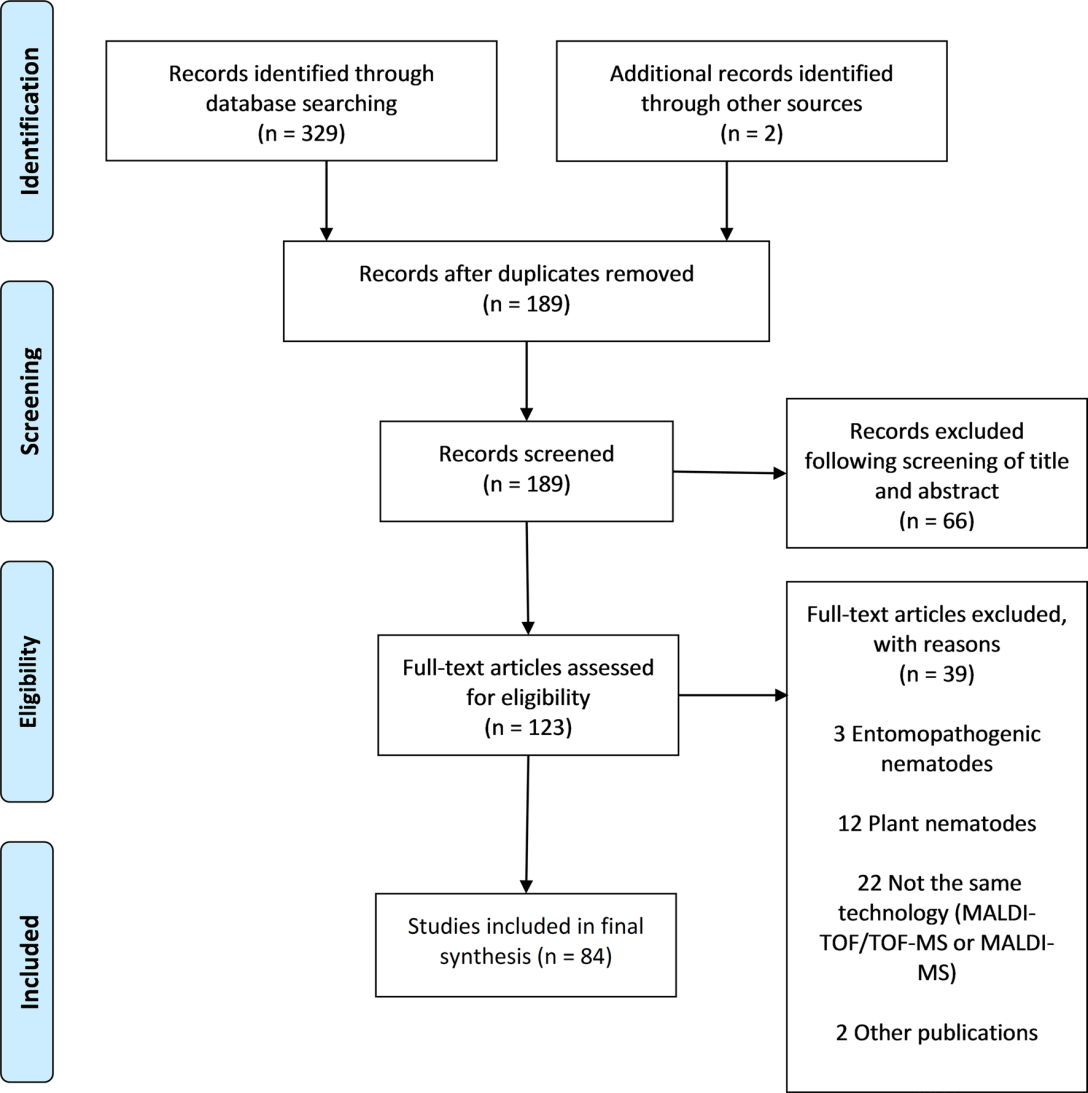
PRISMA diagram for a systematic review examining the application of MALDI-TOF mass spectrometry as potential tool in diagnostic human and veterinary helminthology

**Fig. 2:**
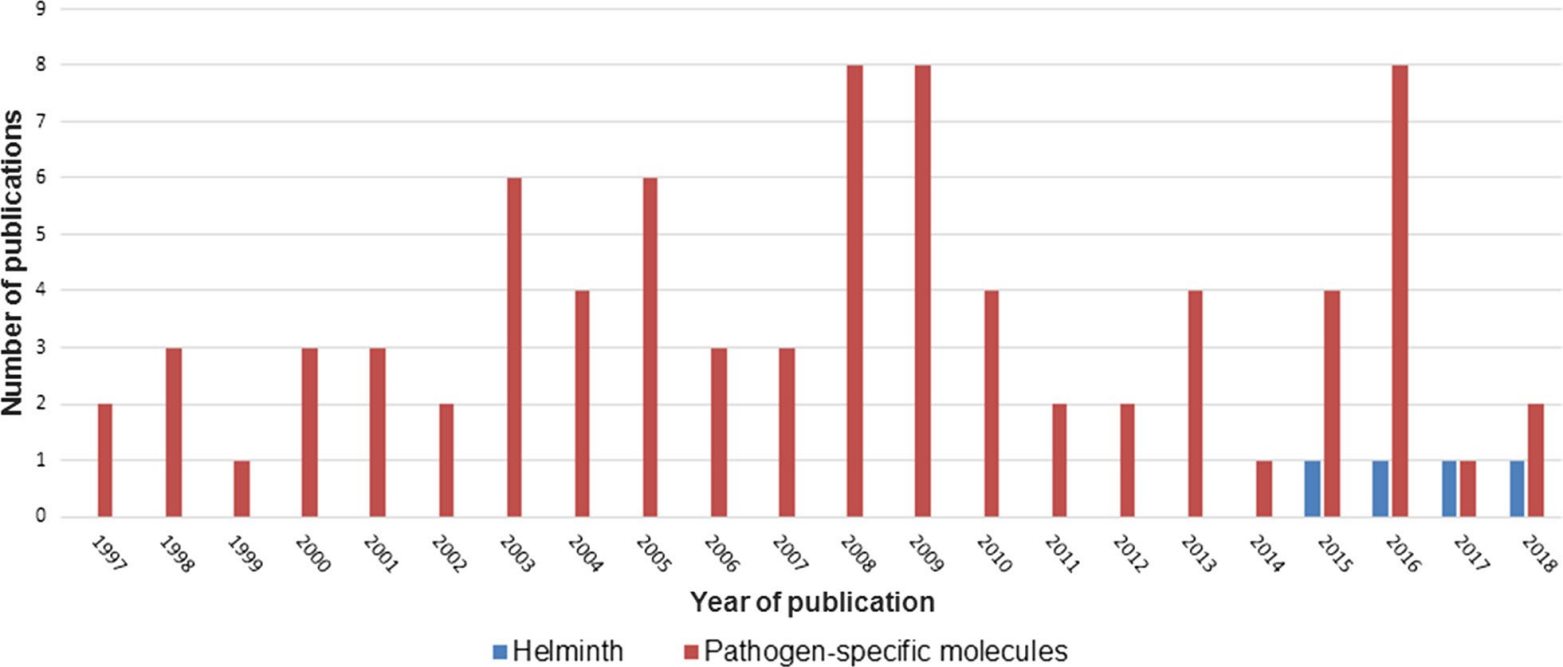
Publications in the peer-reviewed literature pertaining to the application of MALDI-TOF mass spectrometry for identification of helminths or specific pathogen-related components, as revealed by a systematic review, stratified by year of publication

The search procedure and results obtained are shown in Fig. 1. In brief, the initial literature search yielded 329 published studies, with an additional two abstracts identified through further search. Following removal of 142 duplicates, a total of 189 articles were assessed in more detail, of which 66 studies were excluded based on the analysis of the respective titles and abstracts. A full-text analysis was carried out on the remaining 123 studies; 39 articles were finally excluded because their scope was outside the current research question. Hence, 84 articles were included, and these were published between 1997 and 2018. Figure 2 shows the number of publications, stratified by year of publication. The heterogeneity of data reported in the articles precluded any meaningful meta-analysis (Additional file 3).

### Specific applications of MALDI-TOF MS

The first two manuscripts published in 1997 described structural analyses of glycosphingolipids found in *Ascaris suum* and *C. elegans* [63, 64]. Indeed, 95% of all eligible studies used MALDI-TOF MS for identification of specific components rather than for the identification of entire pathogens (Fig. 2). It was only in 2015 when a report on MALDI-TOF MS as diagnostic tool for direct identification of *Dirofilaria* spp. became available [65]. Soon thereafter followed a proof-of-concept study utilizing MALDI-TOF MS for identification and differentiation of *Trichinella* spp. and some narrative reviews mentioning the lack of data on MALDI-TOF in helminthology [32, 66, 67]. Yet, most studies focused on distinct analyses of specific components, such as peptides [66–86], proteins [69, 87–114], lipids [61, 62, 115–124], carbohydrates [125–143] and nucleic acids [144] in a research context. Hence, MALDI-TOF was mainly applied to study and compare the proteome or the peptidome of different helminth species, and most reports focused on *C. elegans*. For example, Husson et al. [74] employed a new approach combining liquid chromatography with MALDI-TOF MS to map and differentiate the neuropeptide profiles of *C. elegans* and the closely related species *C. briggsae*.

The two studies aiming at an identification of entire pathogens provided evidence that MALDI-TOF MS could reliably differentiate between species within the genus *Trichinella* [67] and *Dirofilaria* [65], respectively. In the study by Mayer-Scholl et al. [67], nine species and three genotypes of *Trichinella* isolated from mice, domestic pigs, wild boars and guinea pigs were utilized to create an in-house database with 27 raw spectra generated per specimen. All tested isolates could be distinguished with high diagnostic accuracy. The study by Pshenichnaya et al. [65], which had only been published as a conference abstract, investigated five *Dirofilaria repens* and five *D. immitis* specimens, the causative agents of human and veterinary dirofilariasis, and reported that these could be well differentiated by MALDI-TOF MS. However, data were limited regarding the origin of the study samples, the quality of the spectra obtained by MALDI-TOF and the repeatability of the results. Yet, during the revision of this systematic review, Pshenichnaya et al. [145] published their work on dirofilariasis in a peer-reviewed journal and provided also data for two different species of *Ascaris* (i.e. *A. suum* and *A. lumbricoides*). These helminths could be differentiated by MALDI-TOF based on specific peaks and protein spectra patterns after a cell lysis using the Sepsityper Kit 50 (Bruker Daltonics; Bremen, Germany) and a protein extraction with 70% formic acid and acetonitrile. However, this study has several limitations, and it remains unclear whether calibration steps or assessments of the repeatability and reproducibility of the analyses were performed. An additional paper, published in 2017, reported on MALDI-TOF MS application for cyathostomin helminths, a very diverse group of intestinal parasites infecting horses [66]. These so-called “small strongyles” show a high degree of resistance against benzimidazole anthelminthics and may lead to severe equine enteropathy, colic and death [146]. The study examined several species belonging to the cyathostomin helminths (e.g. *Coronocyclus coronatus*, *C. labiatus* and *C. labratus*) and found distinct protein spectra among adult helminths of different species [66]. These findings were recently confirmed and substantiated by another study on the application of MALDI-TOF for differentiation of cyathostomins, which was published in April 2019 [147].

## Discussion

We systematically reviewed the available literature pertaining to the application of MALDI-TOF MS for identification of helminthic pathogens of human and veterinary importance. While the technique has been successfully employed for many major classes of pathogens (e.g. bacteria, mycobacteria and fungi), data on its use in diagnostic helminthology are scarce. Several studies reported on the differential analysis of specific components, such as proteins, peptides or lipids with MALDI-TOF MS techniques, but only two recent manuscripts and one conference abstract provided ‘proof-of-concept’ evidence of its potential utility in diagnosing and differentiating helminth species of medical or veterinary relevance.

The majority of articles identified in this systematic review focused on protein-centred analyses of helminth samples. It is important to mention that some of the MALDI-TOF MS devices employed in these studies had been subjected to modifications that are not usually available in routine clinical laboratories. Additionally, these experiments frequently employed a complex sample pre-treatment comprising a protein separation by high pressure liquid chromatography (HPLC) or electrophoresis. Yet, some recent proof-of-concept studies have shown that MALDI-TOF MS is also capable of diagnosing entire helminthic pathogens and differentiating similar species within the same genus based on an analysis of their individual protein spectra [66, 67]. Because no helminths are currently included in commercially available MALDI-TOF MS identification databases, individual in-house databases need to be created through generation of main spectra libraries, ideally following established guidelines and protocols that are similar to those employed by the manufacturers of commercially available mass spectrometers [148]. Indeed, previous studies have described the sensitive, reliable and highly reproducible identification of helminths that cause plant infections and have concluded that MALDI-TOF MS should be more widely employed as a ‘rapid detection tool’ [54–58]. Ahmad et al. [56], for example, reported on the suitability of MALDI-TOF MS to differentiate harmless and juvenile infective stages of single plant nematodes, as these showed unique, characteristic protein peak patterns. These studies should be considered as relevant because plant-parasitic nematodes can sometimes also be found in human stool samples [149, 150]. In Brazil, for example, eggs of the root-knot nematode *Meloidogyne* spp. were detected in human faeces using a microscopic sedimentation method [151]. Future studies should also employ MALDI-TOF on serum, as a recent study reported the detection of specific proteins in serum of mice infected with *Schistosoma japonicum* [50].

While helminth infections pose a considerable burden on human and animal health [152], an accurate diagnosis of these conditions is frequently challenging. Indeed, simple diagnostic tools such as stool microscopy for soil-transmitted helminth infections are of limited value if the infection intensity is low and highly sensitive diagnostic techniques such as PCR-based assays are only available in selected reference laboratories outside endemic areas [153]. In high-income countries, in contrast, knowledge regarding standard diagnostic parasitology is waning and differentiation of closely related helminth species based on their microscopic morphology requires skilled laboratory technicians [154]. Moreover, some infections cannot be reliably distinguished with standard diagnostic techniques. A prominent example are infections caused by cestodes of the genus *Taenia* [155], which may cause a relatively harmless intestinal infection if cysts of *Taenia saginata* or *T. solium* are orally ingested with meat of cattle or pig. While eggs of *T. saginata* are not infectious to humans, *T. solium* eggs can lead to the potentially fatal disease (neuro-)cysticercosis. While the correct diagnosis has important implications for treatment, patient management and potential contact screening (intestinal carriage of adult *T. solium* worms poses an increased risk of cysticercosis for close contacts, such as family members), it is impossible to distinguish both species based on the identical morphology of their eggs under a microscope. Molecular tools can achieve an accurate differentiation of the two species, but are only available in research settings [155–157]. Sometimes, proglottids of adult worms are also passed in the faeces. While a distinct differentiation is possible based on the uterine branches within a proglottid, misidentification using this approach has been reported in clinical practice [158]. Hence, achieving a species-specific differentiation based on MALDI-TOF MS would contribute to an enhanced, more reliable identification, and future studies should thus address this issue. Similar considerations hold also true for other infective agents that can hardly be differentiated by other methods (e.g. different *Echinococcus* species), novel species (e.g. hybrid species of *Schistosoma* spp., which have recently been reported from Corsica, France [159]) and notoriously difficult-to-detect infections (e.g. strongyloidiasis). An overview of pathogens for which development of MALDI-TOF MS identification protocols would appear particularly promising is summarized in Table 1.

**Table 1:** Current parasitological techniques, related challenges and research needs for a potential application of MALDI-TOF MS as diagnostic tool for major helminths of human and veterinary importance

Characteristics	Nematodes		Cestodes		Trematodes
Helminth species	Soil-transmitted helminths: *Ascaris lumbricoides*; hookworm; *Strongyloides stercoralis*; *Trichuris trichiura*	Tissue nematodes: *Dirofilaria* spp.; *Trichinella* spp.; *Toxocara* spp.; *Wuchereria bancrofti* and other agents causing lymphatic filariasis, *Onchocerca volvulus*, *Loa loa*; animal hookworms causing cutaneous larva migrans	*Taenia* spp.; *Diphyllobothrium latum*	*Echinococcus* spp.	*Schistosoma* spp.; *Fasciola hepatica*; small liver flukes
Type of diagnostic sample	(i) Stool; (ii) Excreted worms	(i) Tissue samples; (ii) Serum; (iii) Extracted worms (e.g. *Loa loa*)	(i) Stool; (ii) Serum (for *T. solium*); (iii) Tissue samples in cysticercosis; (iv) Excreted proglottids	(i) Stool (in animals); (ii) Tissue biopsies, surgical samples; (iii) Serology	(i) Stool; (ii) Urine; (iii) Serum; (iv) Tissue biopsies
Parasitological standard diagnostic techniques	(i) Stool microscopy (e.g. Kato-Katz technique); (ii) PCR in reference laboratories	(i) Direct identification on biopsy samples; (ii) Serology; (iii) PCR in reference laboratories; (iv) Detection of microfilariae in blood; (v) Detection of antigen in blood (*Wuchereria*)	(i) Direct identification of faecally excreted proglottids; (ii) Stool microscopy; (iii) Serology	(i) Microscopy; (ii) PCR of cysts and tissue samples; (iii) Serology	(i) Stool/urine microscopy (dependent on infecting species); (ii) Rapid diagnostic test for circulating cathodic antigen (CCA) in urine; (iii) PCR on serum, stool or urine; (iv) Serology
Difficulties related to currently employed diagnostics	(i) Low sensitivity in light infection intensities; (ii) Specific concentration techniques needed for *S. stercoralis*; (iii) Misidentification of hookworm and *S. stercoralis* larvae possible; (iv) No species differentiation between different hookworm species possible upon microscopy	(i) Serology frequently false-negative; (ii) False-negative PCR results in case of ‘new’ species; (iii) Lack of expertise outside specialized laboratories	(i) Eggs of related *Taenia* spp. are indistinguishable by microscopy; (ii) Lack of expertise in proglottid differentiation in many laboratories	(i) Serology frequently false-negative in intact cysts; (ii) Microscopy (similar morphology) and serology (cross-reactivity) cannot reliably distinguish between *Echinococcus* spp. (therapeutic implications)	(i) Low sensitivity in light infection intensities; (ii) Microscopy and PCR fail to detect hybrid species
Research needs for MALDI-TOF application	(i) Establishment of a database for identification of eggs/larvae and adult worms; (ii) Differentiation of microscopically indistinguishable hookworm species; (iii) Protocol development for application on stool samples and bronchial specimens	(i) Establishment of a database for identification of tissue-invasive helminths; (ii) Species differentiation within one genus (e.g. *Dirofilaria*); (iii) Application on biopsy specimens; (iv) Detection of microfilariae in blood; (v) Detection of antigen in blood (e.g. *Wuchereria* spp.)	(i) Establishment of a database for identification of *Taenia* proglottids and eggs; (ii) Differentiation between *T. solium* eggs/proglottids and other *Taenia* spp.; (iii) Protocol development for application on stool specimens	(i) Establishment of a database for species-specific identification of eggs and tissue cysts; (ii) Protocol for application on different sample types	(i) Establishment of a database for species-specific identification; (ii) Protocol for application on different sample types; (iii) Detection of *Schistosoma* antigen(s) in serum; (iv) Detection of products derived from helminth-specific metabolism in serum samples

It is important to consider the fixative in which a parasitological sample is stored. Both formaldehyde and ethanol are commonly used to enable a long-term storage of biological specimens, but this may lead to profound changes of the protein structure [160], which is likely to influence on the results of MALDI-TOF examinations carried out on such samples. The virtual impossibility to amplify nucleic acids from formaldehyde-containing solutions [161] due to fragmentation of the single components [162] renders most PCR tests useless on these sample types, but MALDI-TOF analyses of protein spectra might still be possible, albeit with different spectra if compared to native samples. Hence, future studies should evaluate this technique on different kinds of fixatives and on samples that have been stored for prolonged periods.

The present review identified only a few successful studies that employed MALDI-TOF MS to diagnose helminths. Limitations include the complicated pre-treatment procedures employed in some studies and the rather incomplete data presentation in one of the more clinically oriented research projects [65]. New research is needed to determine whether this technique might become a clinically meaningful addendum to the current set of diagnostic options. However, experiences made in clinical bacteriology, mycobacteriology, mycology as well as with ectoparasites (e.g. ticks) and vectors (e.g. mosquitoes) [12, 37, 163] are promising. Whereas MALDI-TOF MS is mainly used on culture-grown colonies for identification of bacteria and mycobacteria, the goal in helminthology will be to provide a species-specific diagnosis based on either macroscopic elements or eggs and larvae that are present in stool samples (or other body fluids and tissue samples). Hence, specific protocols will need to be elaborated to this end, which may include sample preparation, purification and concentration steps, including guidance on the most appropriate sample preservation. However, such protocols have been successfully developed in the past (e.g. for identification of mycobacteria or moulds) [164, 165]. More recently, specific pre-treatment modifications have even allowed to apply MALDI-TOF MS on blood culture broths [166] and fresh urine samples for direct identification of bacteria [167]. Additionally, detection of parasites in complex samples (e.g. blood), should be considered (e.g. as an antigen test for *Wuchereria bancrofti* [168] or for the detection of specific serum peptides [169]).

Yet, much research and rigorous validation is still needed before MALDI-TOF MS might be employed directly on stool samples, and priority should thus be given to (i) the establishment of in-house main spectra library databases to allow for species-specific identification of selected helminths; (ii) the subsequent development of sample treatment protocols; (iii) the validation of this technique on different clinical sample types; and (iv) the elaboration of MALDI-TOF MS to be employed on fixed samples.

## Conclusions

The present systematic review elucidated that MALDI-TOF MS, which is now routinely used in many clinical microbiology laboratories for identification of bacteria, fungi and mycobacteria, could potentially also be employed in the context of helminth diagnosis. Preliminary data suggest that MALDI-TOF MS might hold promise as a future diagnostic tool for direct and rapid identification of pathogenic helminths in clinical samples with sufficient diagnostic accuracy. Further studies are needed to evaluate these concepts and to develop specific databases for helminth identification, followed by rigorous validation on well characterised clinical specimens.

## Additional files

**Additional file 1.** Search strategies employed for our systematic review pertaining to the application of MALDI-TOF mass spectrometry as a diagnostic tool in human and veterinary helminthology.

**Additional file 2.** PRISMA checklist for a systematic review examining the application of MALDI-TOF mass spectrometry as potential tool in diagnostic human and veterinary helminthology.

**Additional file 3.** List of references included in the final review (*n* = 84 articles).

## Data Availability

The search strategy and all manuscripts included in this systematic review are available within the article and its additional files.

## Abbreviations

DALY: disability-adjusted life year
GBD: Global Burden of Disease (Study)
MALDI-TOF: matrix-assisted laser desorption/ionization time-of-flight mass spectrometry
MS: mass spectrometry
PCR: Polymerase chain reaction
