# Supplementary material for: MALDI-TOF mass spectrometry as a diagnostic tool in human and veterinary helminthology: a systematic review
Source: Parasit Vectors. 2019 May 17;12:245. doi: 10.1186/s13071-019-3493-9 (PMC6525464; doi:10.1186/s13071-019-3493-9)
Supplement: Supplementary file 1 — Additional file 1. Search strategies employed for our systematic review pertaining to the application of MALDI-TOF mass spectrometry as a diagnostic tool in human and veterinary helminthology. [file 13071_2019_3493_MOESM1_ESM.pdf]

Additional file 1: Search strategies employed for our systematic review pertaining to the application of MALDI-TOF mass spectrometry as a diagnostic tool in human and veterinary helminthology

Database: MEDLINE/PubMed 1946 to September 2018
Search strategy: 10 October 2018

#	Searches	Results
#1	(MALDI-TOF[Title/Abstract]) OR Matrix Assisted Laser Desorption Ionisation Time Of Flight [Title/Abstract] OR Matrix Assisted Laser Desorption Ionization Time Of Flight [Title/Abstract])	20674
#2	(Helminth[Title/Abstract] OR Nematode[Title/Abstract] OR Cestode[Title/Abstract] OR Trematode[Title/Abstract] OR Helminths[Title/Abstract] OR Nematodes[Title/Abstract] OR Cestodes[Title/Abstract] OR Trematodes[Title/Abstract] OR Worm[Title/Abstract] OR Worms[Title/Abstract])	76572
#3	(MALDI-TOF[Title/Abstract]) OR Matrix Assisted Laser Desorption Ionisation Time Of Flight [Title/Abstract] OR Matrix Assisted Laser Desorption Ionization Time Of Flight [Title/Abstract]) AND (Helminth[Title/Abstract] OR Nematode[Title/Abstract] OR Cestode[Title/Abstract] OR Trematode[Title/Abstract] OR Helminths[Title/Abstract] OR Nematodes[Title/Abstract] OR Cestodes[Title/Abstract] OR Trematodes[Title/Abstract] OR Worm[Title/Abstract] OR Worms[Title/Abstract])	120

Database: ScienceDirect-Embase 1947 to September 2018
Search strategy: 10 October 2018

#	Searches	Results
1	'MALDI-TOF' OR 'Matrix Assisted Laser Desorption Ionisation Time Of Flight' OR 'Matrix Assisted Laser Desorption Ionization Time Of Flight'	8331
2	'helminth' OR 'helminths' OR 'nematode' OR 'nematodes' OR 'cestode' OR 'cestodes' OR 'trematode' OR 'trematodes' OR 'worm' OR 'worms'	29663
3	('MALDI-TOF' OR 'Matrix Assisted Laser Desorption Ionisation Time Of Flight' OR 'Matrix Assisted Lase) AND ('helminth' OR 'helminths' OR 'nematode' OR 'nematodes' OR 'cestode' OR 'cestodes' OR 'trematode' OR 'trematodes' OR 'worm' OR 'worms')	28

Database: Cochrane Library, September 2018
Search strategy: 10 October 2018

#	Searches	Results
#1	(Matrix Assisted Laser Desorption Ionisation Time Of Flight):ti,ab,kw OR (Matrix Assisted Laser Desorption Ionization Time Of Flight):ti,ab,kw OR (MALDI-TOF):ti,ab,kw	150
#2	("helminth"):ti,ab,kw OR ("nematode"):ti,ab,kw OR ("trematode"):ti,ab,kw OR ("cestode"):ti,ab,kw OR (helminths):ti,ab,kw	574
#3	#1 AND #2	0

Database: Web of Science 1975 to September 2018
Search strategy: 10 October 2018

#	Searches	Results
#1	TS= (MALDI-TOF OR Matrix assisted laser desorption ionisation OR Matrix assisted laser desorption ionzsation)	35263
#2	TS=(helminth OR helminths OR nematode OR nematodes OR cestode OR cestodes OR trematode OR trematodes OR worm OR worms)	115459
#3	#2 AND #1	180

Database: Google Scholar (without patents and citations)
Search strategy: 10 October 2018

#	Searches	Results
1	allintitle: 'Nematode' OR 'Helminth' OR 'Cestode' OR 'Trematode' OR 'Nematodes' OR 'Helminths' OR 'Cestodes' OR 'Trematodes' OR 'Worms' OR 'Worm'	186 00 0
	allintitle: 'MALDI-TOF'	8580
2	allintitle: "'Matrix Assisted Laser Desorption Ionization Time Of Flight' 'Matrix Assisted Laser Desorption Ionisation Time Of Flight'"	2
3	allintitle: 'Nematode' OR 'Helminth' OR 'Cestode' OR 'Trematode' OR 'Nematodes' OR 'Helminths' OR 'Cestodes' OR 'Trematodes' OR 'Worms' OR 'Worm' AND 'MALDI-TOF'	1
4	allintitle: 'Nematode' OR 'Helminth' OR 'Cestode' OR 'Trematode' OR 'Nematodes' OR 'Helminths' OR 'Cestodes' OR 'Trematodes' OR 'Worms' OR 'Worm' AND 'Matrix Assisted Laser Desorption Ionization Time Of Flight'	0
	Total	1

TOTAL: 329 PUBLICATIONS
